# Rejuvenating the Brain With Chronic Exercise Through Adult Neurogenesis

**DOI:** 10.3389/fnins.2019.01000

**Published:** 2019-09-18

**Authors:** Mariela F. Trinchero, Magalí Herrero, Alejandro F. Schinder

**Affiliations:** Laboratorio de Plasticidad Neuronal, Fundación Instituto Leloir, Buenos Aires, Argentina

**Keywords:** aging, synaptogenesis, hippocampus, circuit remodeling, neurodegeneration

## Abstract

The aging brain presents a general decline in plasticity that also affects hippocampal neurogenesis. Besides the well-known reduction in the rate of neuronal generation, development of new neurons is largely delayed in the aging brain. We have recently shown that this slow development is accelerated when middle-aged mice perform voluntary exercise in a running wheel. It is unclear whether the effects of exercise on neurogenic plasticity are persistent in time in a manner that might influence neuronal cohorts generated over an extended time span. To clarify these issues, we examined the effects of exercise length in 3-week-old neurons and found that their development is accelerated only when running occurs for long (3–4 weeks) but not short periods (1 week). Furthermore, chronic running acted with similar efficiency on neurons that were born at the onset, within, or at the end of the exercise period, lasting until 3 months. Interestingly, no effects were observed on neurons born 1 month after exercise had ended. Our results indicate that multiple neuronal cohorts born throughout the exercise span integrate very rapidly in the aging brain, such that the effects of running will accumulate and expand network assembly promoted by neurogenesis. These networks are likely to be more complex than those assembled in a sedentary mouse due to the faster and more efficient integration of new neurons.

## Introduction

The generation of new neurons in the adult hippocampus, a region of the brain involved in spatial navigation and memory formation (Buzsaki and Moser, 2013), is a striking form of plasticity that persists throughout life in several species including humans (Altman and Das, 1965; Eriksson et al., 1998; Moreno-Jiménez et al., 2019). Among the many conditions regulating adult hippocampal neurogenesis, one of the most reliable processes that has been widely described is the age-mediated decline in neuronal production, while preexisting neuronal populations remain constant with normal aging (Kuhn et al., 1996; Kempermann et al., 1998; Burke and Barnes, 2010). Aging affects many functions in the brain including synaptic transmission and plasticity, which are thought to contribute to memory loss (Burke and Barnes, 2006; Fan et al., 2017). Given that the hippocampus is particularly vulnerable to age-related alterations and neurodegeneration, finding strategies to enhance plasticity in this structure becomes relevant to prevent or alleviate the effects of senescence (Bartsch and Wulff, 2015). Physical exercise and cognitive stimuli enhance brain health and tend to ameliorate the effects of aging. One of the direct benefits may arise from the activity-dependent increase in the levels of brain-derived neurotrophic factor (BDNF), which has been shown to restore synaptic plasticity, enhance neurogenesis, and improve learning in middle-aged mice (Marlatt et al., 2012; Trinchero et al., 2017). Exercise also reduces microglia activation, increases activity-dependent synaptic plasticity, proliferation of neural progenitor cells and accelerates development and integration of adult-born granule cells (GCs) in the aging hippocampus (van Praag et al., 2005; Kronenberg et al., 2006; O’Callaghan et al., 2009; Marlatt et al., 2012; Gebara et al., 2013). These effects contribute to restore hippocampal-dependent plasticity and correlate with adaptive behavior. Thus, aged animals that perform poorly in spatial learning and pattern separation tasks, improve their performance after exercise (van Praag et al., 2005; Marlatt et al., 2012; Wu et al., 2015; Duzel et al., 2016; Xu et al., 2017).

The extent to which neurogenesis contributes to the positive behavioral effects of exercise in aging animals is still under scrutiny. It is possible that the increase in the rate of neurogenesis exerted by physical exercise contributes to behavioral improvement. But other mechanisms may be implicated as well (Meshi et al., 2006). The accelerated integration of new neurons in the aging dentate gyrus after exercise, which modifies the quality of new GCs, may also contribute to behavioral improvement. While neurons born in middle-aged mice develop slowly, sustained voluntary exercise promotes dendritic growth, spine formation and neuronal integration (Trinchero et al., 2017). Similar effects were observed by brief exposures to environmental enrichment (EE) (Trinchero et al., 2019). If accumulated over multiple neuronal cohorts, the accelerated neuronal integration triggered by exercise might exert significant influence on hippocampus-dependent learning and behavior. In this work we demonstrate that several cohorts of new GCs can be rapidly integrated in the preexisting circuits of mice that continue to engage in voluntary running, and that these effects persist once mice have stopped running.

## Materials and Methods

### Mice and Surgery

C57BL/6J male mice were housed at 4–5 animals per cage under standard conditions. Eight-month-old (8M) mice were selected because, beyond this age, there is a strong decline in hippocampal neurogenesis that precludes the study of labeled neurons (Morgenstern et al., 2008; Trinchero et al., 2017). Mice were anesthetized (150 μg ketamine/15 μg xylazine in 10 μl saline/g), and retrovirus was infused into the septal region of the right dentate gyrus (1.5 μl at 0.15 μl/min) using sterile calibrated microcapillary pipettes through stereotaxic surgery; coordinates from bregma (in mm): −2 anteroposterior, −1.5 lateral, and −1.9 ventral. At the indicated times, brains were fixed and sections were prepared for confocal imaging (Trinchero et al., 2017). Only GCs from the septal dentate gyrus were included in the analysis, corresponding to sections localized from −0.96 to −2.30 mm from the bregma, according to the mouse brain atlas (Paxinos and Franklin, 2001). Experimental protocols were approved by the Institutional Animal Care and Use Committee of Fundación Instituto Leloir, according to the Principles for Biomedical Research involving animals of the Council for International Organizations for Medical Sciences and provisions stated in the Guide for the Care and Use of Laboratory Animals.

### Running

**FIGURE 1:**
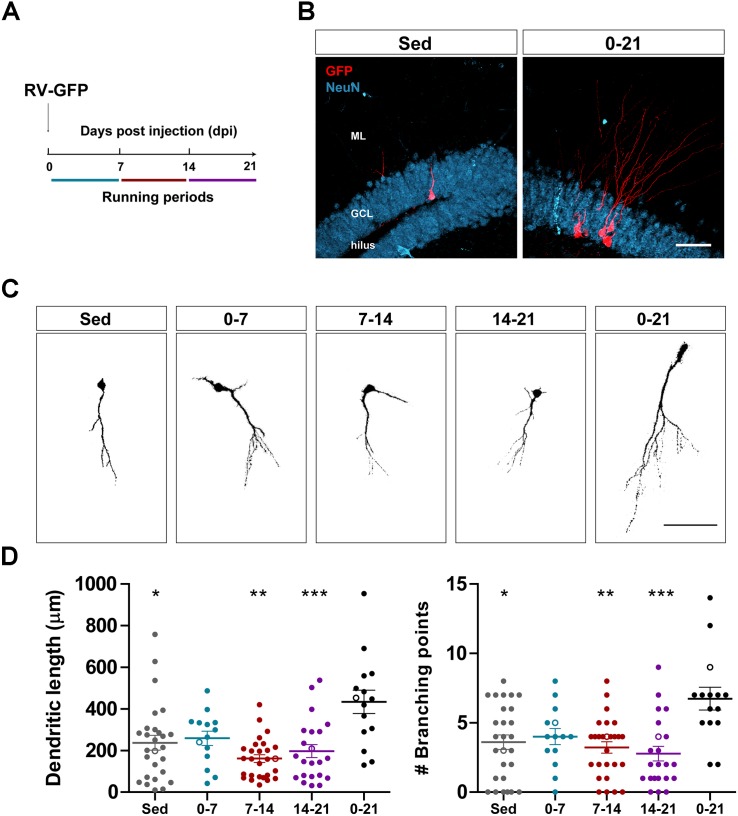
Long- but not short periods of running accelerate morphological maturation of new GCs. **(A)** Experimental design. RV-GFP injection was followed by exposure to 1 week of voluntary running at the indicated windows or for the entire experiment (0–21 dpi). **(B)** Representative images of 21-dpi GCs expressing GFP (red) taken from sedentary and 0–21 running groups. NeuN (blue) labels the granule cell layer (GCL) (ML, molecular layer). Scale bar, 50 μm. **(C)** Representative confocal images of 21-dpi GFP-GCs for the different groups. Scale bar, 50 μm. **(D)** Dendritic complexity (length and branching points) for the different running windows. ^∗^, ^∗∗^, and ^∗∗∗^ denote *p* < 0.05, *p* < 0.01, and *p* < 0.001 compared to the 0–21 running group after Kruskal–Wallis test followed by Dunn’s *post hoc* test. No differences were found among any of the groups running for 7 days. Sample sizes (neurons/mice): 27/3 (Sed), 14/3 (0–7), 27/3 (7–14), 22/3 (14–21), and 15/3 (0–21). Horizontal bars denote mean ± SEM. Open circles correspond to example neurons.

**FIGURE 2:**
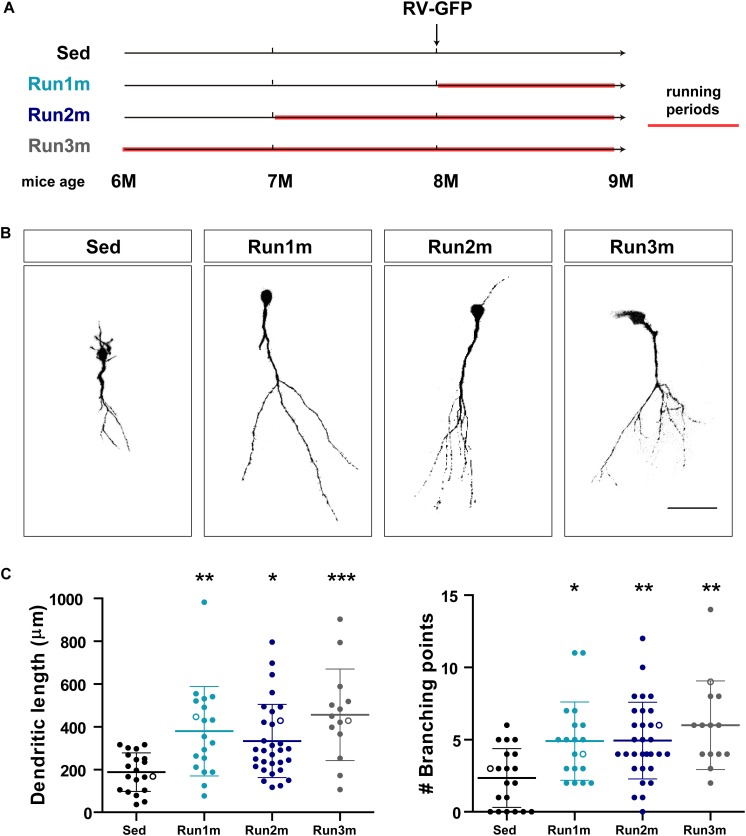
Effects of running on different neuronal cohorts. **(A)** Experimental design. RV-GFP injection was followed by 3 weeks of running and preceded by sedentary conditions (Run1m), 1 (Run2m), or 2 months of running (Run3m). All groups were compared with sedentary mice (Sed). Total dendritic length was analyzed at 21 dpi. **(B)** Representative confocal images GFP-GCs. Scale bar, 50 μm. **(C)** Dendritic complexity (length and branching points) for the different windows of running. ^∗^, ^∗∗^, and ^∗∗∗^ denote *p* < 0.05, *p* < 0.01, and *p* < 0.001 compared to Sed after Kruskal–Wallis test followed by Dunn’s *post hoc* test. Sample sizes (neurons/mice): 20/3 (Sed), 19/3 (Run1m), 31/3 (Run2m), and 15/3 (Run3m). Horizontal bars denote mean ± SEM. Open circles correspond to example neurons.

**FIGURE 3:**
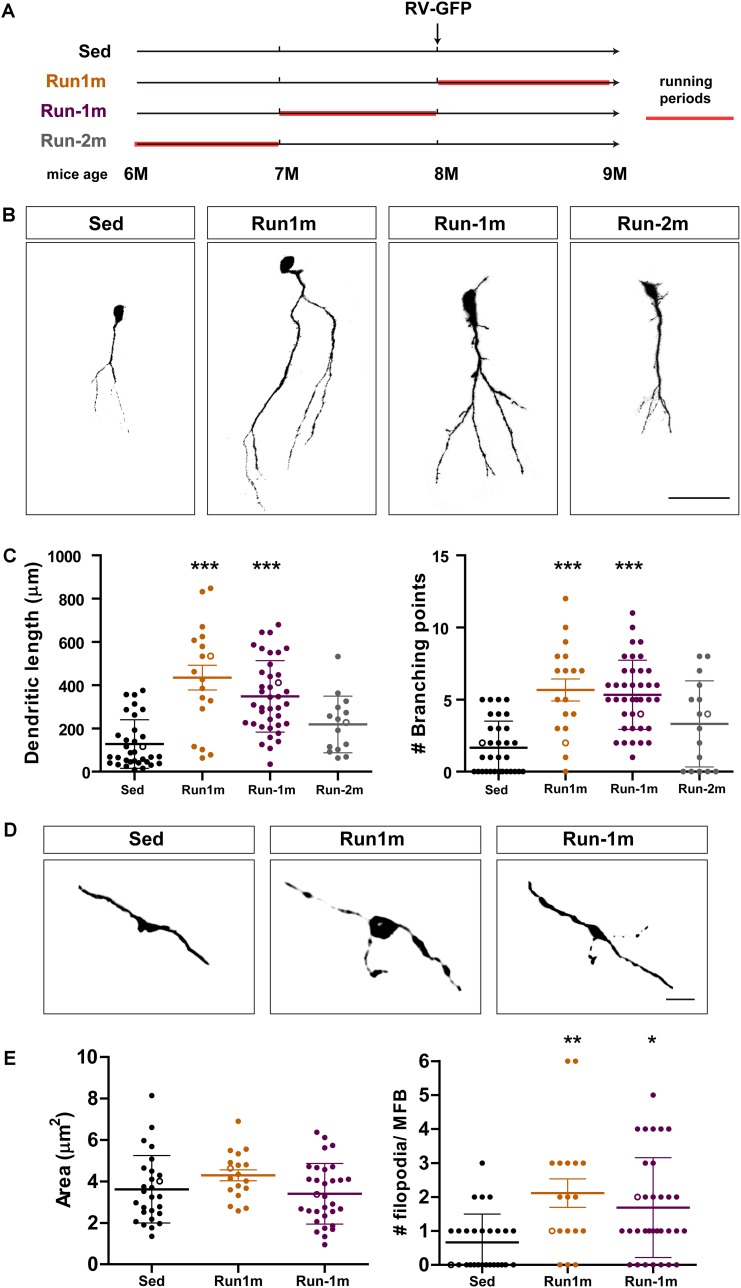
Persistent effects of chronic exercise. **(A)** Experimental design. RV-GFP injection was followed by 3 weeks of running (Run1m) or preceded by 1 month of exercise (Run-1m) or 1 month of exercise and 1 month without the running wheel (Run-2m). All groups were compared with sedentary mice (Sed). Total dendritic length was analyzed at 21 dpi. **(B)** Representative confocal images of labeled GCs. Scale bar, 50 μm. **(C)** Dendritic complexity (length and branching points) for the different running windows. ^∗∗∗^ denotes *p* < 0.001 compared to Sed after Kruskal–Wallis test followed by Dunn’s *post hoc* test. Sample sizes (neurons/mice): 33/4 (Sed), 39/4 (Run1m), 15/4 (Run-1m), and 18/3 (Run-2m). **(D)** MFB morphology in CA3 was analyzed for Run1m and Run-1m groups and compared to Sed. Representative confocal images. Scale bar, 5 μm. **(E)** ^∗^ and ^∗∗^ denote *p* < 0.05 and *p* < 0.01 after Kruskal–Wallis test followed by Dunn’s *post hoc* test. Sample sizes: 27/4 (Sed), 32/4 (Run1m), and 18/4 (Run-1m). Horizontal bars denote mean ± SEM. Open circles correspond to example boutons.

Running distances were recorded for each experiment using wireless running wheels. In the experiments of Figure 1 animals were housed with a running wheel for 7 or 21 days, as indicated. In this condition mice ran ∼2 km/day. In Figures 2, 3 in which mice ran for 1, 2, or 3 months, the monitored distance was ∼10 km/day.

### Immunofluorescence

Immunostaining was done on 60-μm free-floating coronal sections. Antibodies were applied in tris-buffered saline (TBS) with 3% donkey serum and 0.25% Triton X-100. Immunofluorescence was performed using anti GFP (rabbit polyclonal; 1:500; Invitrogen), anti NeuN (mouse monoclonal; 1:50; a gift from F.H. Gage, Salk Institute for Biological Studies, La Jolla, CA, United States), donkey anti-rabbit Cy3 and donkey anti-mouse Cy5 antibodies (1:250; Jackson Immuno Research Laboratories).

### Confocal Microscopy

For dendritic length measurements, images were acquired (40×; NA 1.3; oil-immersion) from 60-μm thick sections taking Z stacks including 35–50 optical slices, airy unit = 1 at 0.8-μm intervals (Trinchero et al., 2017). Dendritic length was then measured using the LSM Image Browser software from projections of three-dimensional reconstructions onto a single plane in GCs expressing GFP. Images of GFP-labeled MFBs in the CA3 region were acquired at 0.4-μm intervals (63×; NA 1.4; oil-immersion) and a digital zoom of 6. Area and number of filopodia was analyzed from projections of three-dimensional reconstructions onto a single plane. Mossy fiber boutons (MFB) that fit the following criteria were selected for quantification: (i) the diameter of the bouton was >threefold larger than the diameter of the fiber, (ii) the bouton was connected to the mossy fiber on at least one end (Toni et al., 2008). Filopodia were identified as protrusions arising from large mossy terminals (1 μm < length < 20 μm) (Acsady et al., 1998). Filopodial extensions were measured by counting the number of protrusions per terminal. For image capture and analysis of morphological properties, all experimental groups under study were blind to the operator.

### Statistical Analysis

Unless otherwise specified, data are presented as mean ± SEM. Normality was assessed using the Shapiro–Wilks test, D’Agostino-Pearson omnibus test, and Kolmogorov–Smirnov test, with a *p* value of 0.05. When data met normality tests (Gaussian distribution and equal variance), unpaired *t*-test with Welch’s correction or ANOVA with Bonferroni’s *post hoc* test were used as indicated. In cases that did not meet normality, non-parametric tests were used as follows: Mann–Whitney’s test for independent comparisons, and Kruskal–Wallis test for multiple comparisons.

## Results

We have previously shown that running accelerates development and functional integration of new GCs in the aging hippocampus (Trinchero et al., 2017). We now investigated whether this type of plasticity demands a persistent level of activity that accumulates in time, or if shorter periods of running may also trigger faster integration, as previously observed for the exposure to EE (Trinchero et al., 2019). To address this question, new neuronal cohorts were labeled in middle-aged mice (8 months of age; 8M) using a retrovirus expressing GFP (RV-GFP). Mice were allowed to use a running wheel for 1 week within different windows of neuronal development, or to run for the entire interval of neuronal growth (3 weeks; Figures 1A,B). As described before, new GCs displayed slow development in sedentary mice, which resulted in short dendrites with little ramifications at 3 weeks. One-week running did not change the general appearance of new GCs in any of the tested intervals (Figures 1C,D). In contrast, running for the entire 3-week interval resulted in increased dendritic length and branching, consistent with an accelerated neuronal integration.

The observed effects of exercise were tested on a single neuronal cohort, the one that was retrovirally labeled. However, because new GCs are continuously being generated, it is conceivable that GCs born at different moments within the exercise period might also be influenced by the activity. To test this hypothesis, we used a simple strategy to label neuronal cohorts born at different times in regard to exercise initiation by exposing mice to running wheels for different intervals, but always analyzing neuronal structure 3 weeks after retroviral injection (termed 1m for simplicity). Three cohorts were thus compared; one running for 3 weeks, labeled right at the onset of exercise (Run1m), one running for 2 months, labeled 1 month after the onset of exercise (Run2m), and one running 3 months, labeled 2 months after onset of exercise (Run3m) (Figure 2A). We observed a similar degree of neuronal growth in all conditions compared to sedentary mice, which indicates that all neuronal cohorts born in the brain of a middle-aged mouse performing voluntary running will undergo rapid growth and integration (Figures 2B,C). Dendritic trees of GCs in Run3m mice were slightly longer than those from Run1m or Run2m mice, suggesting that promotion of neuronal integration might respond to a mechanism whose effect accumulates in time. Yet, 1 month of running resulted in a near maximal effect on GC development.

We then investigated the effects of exercise in the aging brain on neuronal cohorts born once activity ended. We compared how running for 3–4 weeks influences development of new GCs born right at the onset (Run1m), at the end (Run-1m), or 1 month after the end of exercise (Run-2m; Figure 3A). Surprisingly, neuronal cohorts born at the beginning or at the end of the running period responded similarly (Figures 3B,C). In contrast, cohorts born 1 month after the end of exercise (Run-2m) showed only a subtle effect.

To fully integrate into the circuit, GCs establish glutamatergic excitatory connections onto CA3 pyramidal cells through large MFBs, and recruit GABAergic feedforward inhibition on pyramidal cells via filopodial extensions that arise from those terminals (Acsady et al., 1998; Toni et al., 2008; Sun et al., 2013; Restivo et al., 2015). While confocal analysis did not reveal changes in the area of MFBs of neurons born at Run-1m or Run1m compared to controls, the number of filopodia/MFBs increased by ∼100% (Figures 3D,E). These results indicate that physical exercise specifically increases connectivity from new GCs onto the inhibitory local network (Trinchero et al., 2019).

## Discussion

Adult neurogenesis involves a sequence of complex developmental steps that results in the integration of new information-processing units. Neural stem cells of the subgranular zone leave their quiescent state to become proliferating neural progenitor cells that expand the precursor population (transit amplifying cells) (Bonaguidi et al., 2012). Progenitor cells generate neurons that migrate, develop and integrate into the circuit. During this extended time window there is a marked reduction of the neuronal pool due to apoptotic cell death. Voluntary exercise and EE constitute strong stimuli for boosting adult hippocampal neurogenesis in rodents throughout life (van Praag et al., 1999a, b; Kronenberg et al., 2003, 2006; Wu et al., 2008; Kannangara et al., 2011). Neurogenesis declines in the aging brain, mainly as a consequence of the reduction in the size of the progenitor cell pool. Both running and EE can counteract the decreased neuronal production reported in aging animals, acting through different mechanisms (Kempermann et al., 2010). Running acts at two levels to enhance neurogenesis: increasing the rate of proliferation of neural progenitor cells (van Praag et al., 1999b, 2005; Cooper et al., 2018), and accelerating the maturation and functional integration of developing neurons (Trinchero et al., 2017). EE increases survival and also accelerates maturation of new GCs, but it does not influence proliferation (Trinchero et al., 2017, 2019).

Running also promotes rewiring of neuronal connectivity and modulation of intrinsic properties in new GCs during the first week of development in young-adult mice (Sah et al., 2017). We have recently reported a critical period during the second week of GCs maturation in aging animals, in which EE induces dendritic growth and faster integration of new neurons into the circuit (Trinchero et al., 2019). We asked here if running for 1 week at any time of GCs development would affect their integration, but found no effect (Figure 1). The entire 3-week running period was required to regulate neurogenesis. The lack of short-term modulation suggests a mechanism mediated by factors that need to build-up to exert noticeable changes. Interestingly, voluntary exercise exacerbate the production of BDNF, which accumulates over weeks before reaching a *plateau* level that exerts maximal actions (Vecchio et al., 2018). Even though neurotrophins are key mediators, the effects observed here are plausible to be mediated by multiple mechanisms. Exercise also boosts electrical activity in the dentate gyrus, rises blood flow, and modulates neuroinflammation (Piatti et al., 2011; Speisman et al., 2013; Trinchero et al., 2017). The contribution of other mechanisms requires further studies.

We show here that ∼3 week-old GCs from aging mice chronically exposed to a running wheel (up to 3 months) present a fully developed phenotype with long and complex dendritic arborizations, similar to those of new GCs generated in young adult mice (Figure 2). Mature GCs in young-adult and middle-aged mice reach equivalent dendritic length and complexity by the end of development, comparable to 3-week-old GCs in aging mice exposed to EE or running (Piatti et al., 2011; Trinchero et al., 2017, 2019). This accumulated evidence speaks for a clear acceleration in the speed of maturation evoked by running.

At the level of neuronal output, running promoted the rapid growth of filopodial extensions but did not modify the size of MBFs (Figures 3D,E). This result suggests that new GCs integrate rapidly, but their output might be biased toward the activation of GABAergic interneurons in CA3 (contacted by filopodia), rather than pyramidal cells contacted by MFBs (Acsady et al., 1998; Toni et al., 2008; Sun et al., 2013; Restivo et al., 2015). It is surprising that EE provoked a marked increase in both the size of MFBs and the number of filopodial extensions in aging mice, consistent with a more balanced modulation of neuronal output (Trinchero et al., 2019). We speculate that changes due to EE involve activity-dependent remodeling of specific synapses that become strengthened for encoding spatial cues (Nicoll and Schmitz, 2005; Holtmaat and Svoboda, 2009). In contrast, running may represent a general stimulus for new GCs to become prepared to connect to specific targets when encoding becomes necessary, while conserving the inhibitory tone from GABAergic interneurons that might act as a mechanism limiting postsynaptic activity until the new GC has been properly assembled within the surrounding network.

**FIGURE 4:**
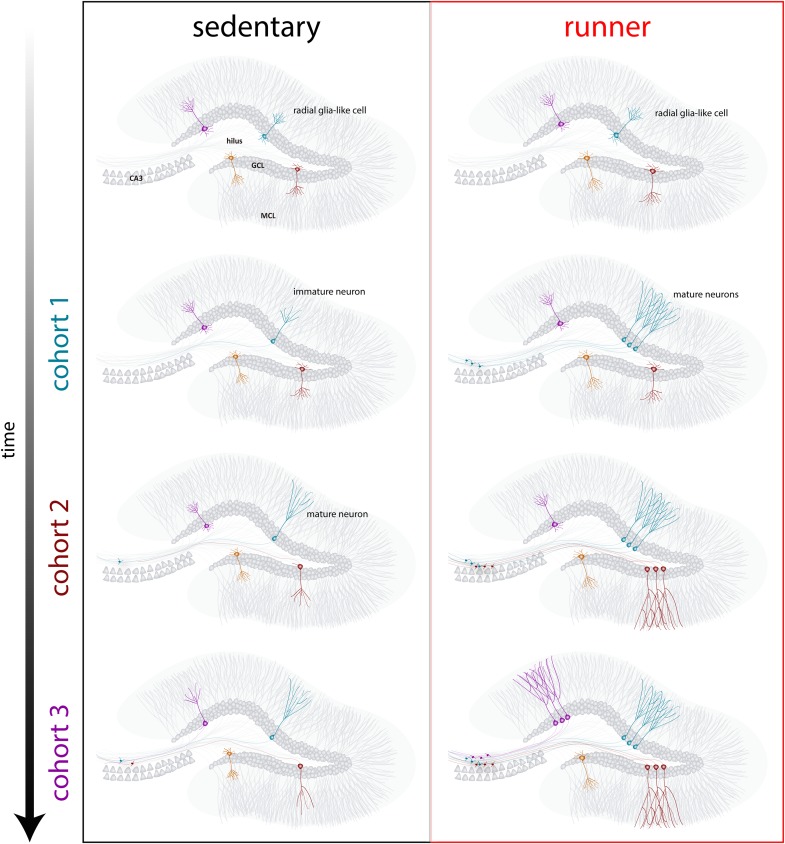
Running-induced rejuvenation of the hippocampal network in middle-aged mice. Schematic representation of the time course of neuronal integration in sedentary (left) and running (right) mice after prolonged intervals of voluntary exercise. In the subgranular zone of the dentate gyrus, radial-glia like cells (RGL) remain in a quiescent state (top). Sedentary aging mice present low rates of neurogenesis and new neurons develop in a slow manner (representative cohorts are indicated with different colors). In running mice, the production of new GCs is boosted, and these new units develop and integrate faster, expanding the neural network in a cumulative manner over an extended time span. Ultimately, these new cohorts of fully mature neurons become engaged to process information upon demand.

Our results indicate that the aging brain generates neurogenic signals when mice continue to run for prolonged periods (months). In this context, subsequent neuronal cohorts born in running mice will develop and integrate rapidly, allowing aging circuits to accumulate substantial numbers of new neurons (Figure 4). The continuous incorporation of new GCs with features that resemble what occurs in younger mice results in a rejuvenated hippocampus with neurons that could be primed to respond to future experiences.

From a broader perspective, the increase in longevity associated to the modern society is accompanied by a higher risk for cognitive decline. Thus, it becomes relevant to understand the mechanisms underlying the well-known benefits of exercise in brain function. In addition, there might be long-term differences among individuals practicing exercise during different intervals in life and those that have performed exercise continuously, in terms of decreasing the risk for developing neurodegenerative diseases (Llorens-Martin, 2018). Our results demonstrate that continuous running promotes the integration of many neuronal cohorts, thus elevating the complexity of the network in a remarkable manner. Designing different strategies for increasing the efficacy of new neuron integration in the hippocampus might serve as a promising mechanism of plasticity to eventually ameliorate the cognitive decline occurring in the healthy and diseased aging brain. Finally, dissecting the mechanisms underlying the different aspects of such accelerated neuronal integration and development will teach us a lot about the potential for plasticity of neural networks in the aging brain.

## Data Availability

The data that support the findings of this study are available from the corresponding author upon reasonable request.

## Ethics Statement

The animal study was reviewed and approved by committee for the use and care of laboratory animals of the Leloir Institute.

## Author Contributions

MT and MH performed the experiments and analyzed the data. MT, MH, and AS designed the experiments and wrote the manuscript. AS provided financial support.

## Conflict of Interest Statement

The authors declare that the research was conducted in the absence of any commercial or financial relationships that could be construed as a potential conflict of interest.
